# The Global Network Maternal Newborn Health Registry: a multi-national, community-based registry of pregnancy outcomes

**DOI:** 10.1186/1742-4755-12-S2-S1

**Published:** 2015-06-08

**Authors:** Carl L Bose, Melissa Bauserman, Robert L Goldenberg, Shivaprasad S Goudar, Elizabeth M McClure, Omrana Pasha, Waldemar A Carlo, Ana Garces, Janet L Moore, Menachem Miodovnik, Marion Koso-Thomas

**Affiliations:** 1 Department of Pediatrics, Division of Neonatal-Perinatal Medicine, University of North Carolina School of Medicine, Chapel Hill, NC, USA; 2 Department of Obstetrics and Gynecology, Columbia University, New York, NY, USA; 3 Women's and Children's Health Research Unit, KLE University's Jawaharlal Nehru Medical College, Belgaum, India; 4 RTI International, Durham, NC, USA; 5 Department of Community Health Sciences, Aga Khan University, Pakistan; 6 Department of Pediatrics, Division of Neonatology, University of Alabama at Birmingham, School of Medicine, Birmingham, AL, USA; 7 Department of Pediatrics, School of Medicine, San Carlos University, Guatemala City, Guatemala; 8 Center for Research for Mothers and Children, Eunice Kennedy Shriver National Institute of Child Health and Human Development, Bethesda, Maryland, USA

**Keywords:** registry, perinatal mortality, neonatal mortality, stillbirth, maternal mortality

## Abstract

**Background:**

The Global Network for Women's and Children's Health Research (Global Network) supports and conducts clinical trials in resource-limited countries by pairing foreign and U.S. investigators, with the goal of evaluating low-cost, sustainable interventions to improve the health of women and children. Accurate reporting of births, stillbirths, neonatal deaths, maternal mortality, and measures of obstetric and neonatal care is critical to efforts to discover strategies for improving pregnancy outcomes in resource-limited settings. Because most of the sites in the Global Network have weak registration within their health care systems, the Global Network developed the Maternal Newborn Health Registry (MNHR), a prospective, population-based registry of pregnancies at the Global Network sites to provide precise data on health outcomes and measures of care.

**Methods:**

Pregnant women are enrolled in the MNHR if they reside in or receive healthcare in designated groups of communities within sites in the Global Network. For each woman, demographic, health characteristics and major outcomes of pregnancy are recorded. Data are recorded at enrollment, the time of delivery and at 42 days postpartum.

**Results:**

From 2010 through 2013 Global Network sites were located in Argentina, Guatemala, Belgaum and Nagpur, India, Pakistan, Kenya, and Zambia. During this period, 283,496 pregnant women were enrolled in the MNHR; this number represented 98.8% of all eligible women. Delivery data were collected for 98.8% of women and 42-day follow-up data for 98.4% of those enrolled. In this supplement, there are a series of manuscripts that use data gathered through the MNHR to report outcomes of these pregnancies.

**Conclusions:**

Developing public policy and improving public health in countries with poor perinatal outcomes is, in part, dependent upon understanding the outcome of every pregnancy. Because the worst pregnancy outcomes typically occur in countries with limited health registration systems and vital records, alternative registration systems may prove to be highly valuable in providing data. The MNHR, an international, multicenter, population-based registry, assesses pregnancy outcomes over time in support of efforts to develop improved perinatal healthcare in resource-limited areas.

Study Registration: The Maternal Newborn Health Registry is registered at Clinicaltrials.gov (ID# NCT01073475).

## Introduction

The Global Network for Women's and Children's Health Research (Global Network) supports and conducts clinical trials in resource-limited countries by pairing foreign and U.S. investigators, with the goal of evaluating low-cost, sustainable interventions to improve the health of women and children. Another goal of the Global Network is to build local research capacity and infrastructure. These activities are designed to strengthen local capacity for independent research that will ultimately contribute to improved health care systems. The Global Network was initiated in 2001 by the *Eunice Kennedy Shriver* National Institute of Child Health and Human Development (NICHD) with co-funding from the Bill & Melinda Gates Foundation.

Accurate reporting of births, stillbirths, neonatal deaths, maternal mortality, and measures of obstetric and neonatal care is critical to efforts to inform health policy and to improve pregnancy outcomes in resource-poor settings. Because most of the study sites in the Global Network are in geographic areas with weak health care systems, they lack precise data on maternal and newborn health outcomes and measures of care. The development of a registry of vital statistics was necessary to allow the Global Network to document maternal and neonatal outcomes, design trials to address the major causes of poor outcomes, and assess interventions to improve outcomes. Collectively, these results will inform public health policy.

The Global Network’s Maternal Newborn Health Registry (MNHR) is a prospective, population-based registry of pregnancies at the Global Network sites. The MNHR began in May 2008, as an expansion of the population-based data collection tool established at sites for the FIRST BREATH Study, a clinical trial investigating the impact of newborn care training on perinatal mortality in resource limited areas in low and low-middle income countries [1,2]. The MNHR continued after the completion of this study. The primary purpose of the MNHR is to quantify and analyze trends in pregnancy outcomes over time in order to provide population-level statistics within defined geographic areas. In this way, the MNHR serves as a data collection tool for pregnancy outcomes in individual studies and provides data to plan future studies in the Global Network. Although the MNHR relies on a core set of data (see below), to support individual studies, investigators may propose time-limited supplemental data collection to the MNHR.

In this supplement, there are a series of manuscripts that use data gathered through the MNHR from 2010 through 2013. During this period, sites in the Global Network remained the same, and a consistent core set of variables was collected. The initial MNHR study sites were located in Argentina, Guatemala, Belgaum and Nagpur, India, Pakistan, Kenya, and Zambia. In 2013, following a competitive renewal at the end of an NICHD funding cycle, the site in Argentina was replaced by a site in the Democratic Republic of Congo. (See Table 1 for locations and principal investigators.) The purpose of this manuscript is to provide an overview of the MNHR; included is a brief description of the sites, its organization and management, and methods of data collection. Selected data describing the subjects enrolled in the MNHR from 2010 to 2013 are included to characterize the population and the perinatal health care. Outcomes during this period are examined in the accompanying manuscripts in this supplement.

**Table 1:** Sites of the Global Network for Women’s and Children’s Health Research

Location of Site	In-country Institution	US Institution	Senior Foreign Principal Investigator	US Principal Investigator
**Corrientes, Argentina**	Institute for Clinical Effectiveness and Health Policy, Buenos Aires	Tulane School of Public Health and Tropical Medicine, New Orleans, LA	Jose Bélizán	Pierre Buekens

Equateur Provence, Democratic Republic of Congo*	Kinshasa School of Public Health, Kinshasa	University of North Carolina-Chapel Hill	Antoinette Tshefu	Carl Bose

**Kafue and Chongwe Provence, Zambia**	University Teaching Hospital, Lusaka	University of Alabama at Birmingham	Elwyn Chomba	Waldemar Carlo

**Chimaltenango, Guatemala**	FANCAP, Fundación para la Alimentación y Nutrición de Centro América y Panamá Guatemala City	University of Colorado School of Medicine	Ana Garces	Nancy Krebs

**Belgaum, Karnataka, India**	Jawaharlal Nehru Medical College, Belgaum	Christiana Care Health Services, Newark, DE	Bhala Kodkany	Richard Derman

**Thatta, Pakistan**	Aga Khan University, Karachi	Columbia University	Omrana Pasha	Robert Goldenberg

**Nagpur, India**	Indira Gandhi Government Medical College, Nagpur	Massachusetts General Hospital for Children	Archana Patel	Patricia Hibberd

**Western Kenya**	Moi University, Eldoret, Kenya	Indiana University School of Medicine	Fabian Esamai	Edward Liechty

**Data Coordinating Center**	RTI International, Durham, NC		Elizabeth McClure	

*Participant in the MNHR since 2013 only.

## Global Network MNHR Sites

The target population for the MNHR at each Global Network site is women who reside in or receive healthcare in a specified group of communities, also referred to as clusters. Each cluster is defined by a geographic area where mothers receive primary perinatal care from designated healthcare facilities. Initially, for a cluster to be included in the MNHR, it was estimated to have 300 to 500 deliveries per year, although the number of annual deliveries has increased in many clusters over time. The clusters usually correspond to existing healthcare service delivery areas, such as an area or zone defined by the ministry of health in the participating country. The clusters are not intended to be representative of the demography or healthcare throughout the country; in general, they represent the resource-limited areas within countries. Each site has established between 10 and 20 research clusters and altogether, the MNHR enrolls approximately 70,000 pregnant women annually.

A brief description of each site follows. These include the Human Development Index (HDI) for each region in which the site is located. The HDI is a composite statistic of life expectancy, education, and income indices used to rank countries. Values are typically >0.850 in Western Europe and North America. In 2014, values worldwide ranged from 0.944 (Norway) to 0.337 (Niger) [3].

## South Asian Sites (Figure 1)

**Figure 1:**
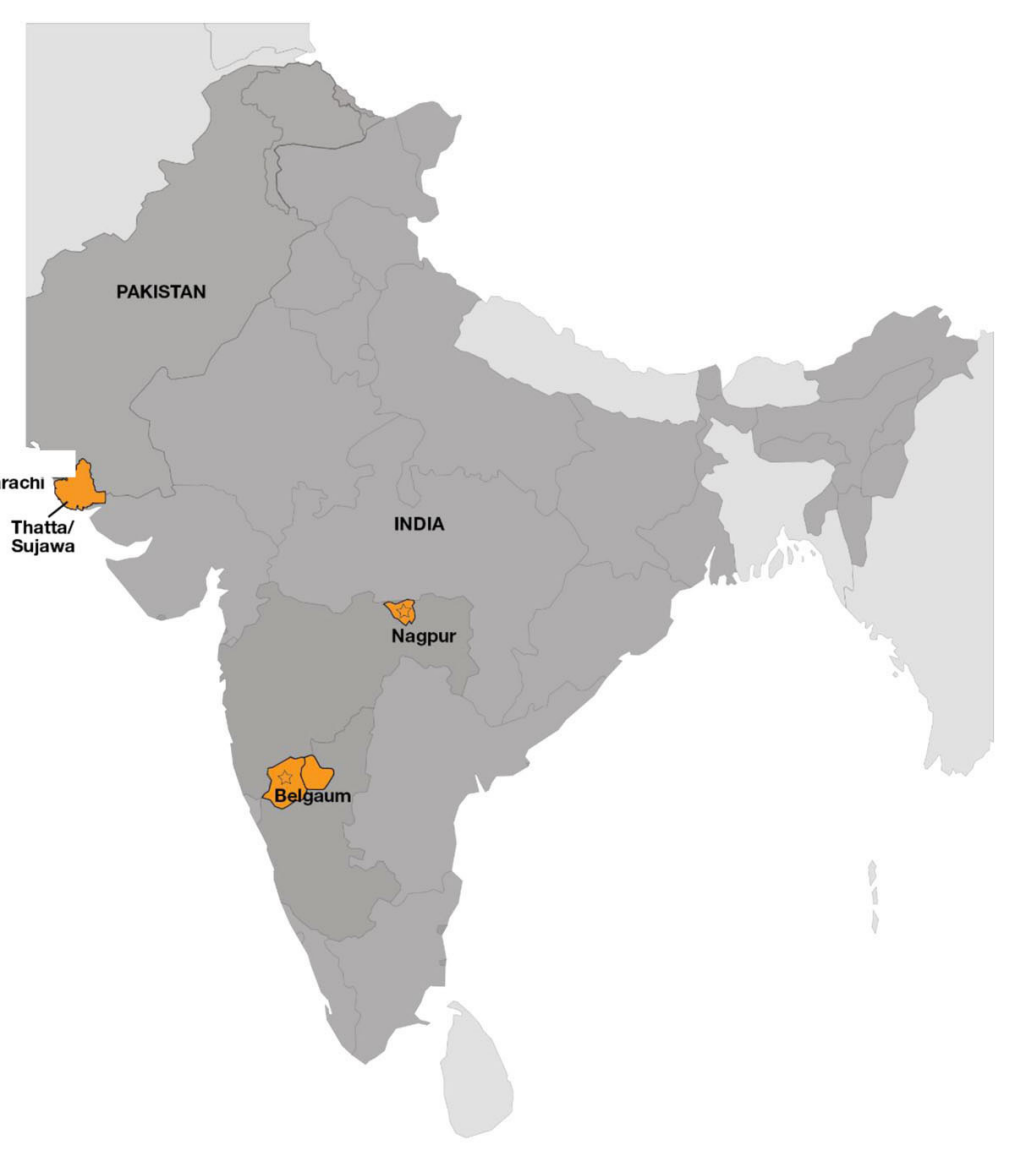
South Asian Global Network Sites

### India (Belgaum)

*Location:* The research site is within the northwestern corner of the southern state of Karnataka, India, with the site coordinating center located in Belgaum.

*Setting:* There are 18 clusters corresponding to the service areas of 18 primary health centers. Each is managed by a physician medical officer who works with nursing staff and auxiliary nurse midwives in associated sub-centers, the most peripheral outpost of the health care services. There are three tertiary care hospitals and eight secondary care hospitals serving the region as referral hospitals staffed by obstetricians, pediatricians and nurses. In addition to these public sector health facilities, there are several private sector maternity facilities within the site catchment area.

*HDI:* 0.508 [6]

### India (Nagpur)

*Location*: The research site is within the state of Maharashtra, India, with the coordinating center in Nagpur.

*Setting*: There are 20 clusters corresponding to the service area of 20 primary health centers, each served by physician medical officers and nurses. These areas include 119 sub-centers where basic maternal and child care are provided. Referral care is provided in ten tertiary hospitals (two public sector and eight private sector), and 129 secondary hospitals (27 public sector hospitals and 102 private nursing homes).

*HDI*: 0.549 [6]

### Pakistan

*Location:* Research sites are in two of five sub-districts within the Thatta district in the southern Sindh province, near the city of Karachi, where the site coordinating center is located.

*Setting:* The 20 clusters are served by over 75 health facilities, both public sector and private fee-for-service, providing maternal and child health services. This includes 47 primary health clinics, 25 secondary care facilities and 3 referral hospitals. Care in health clinics is typically provided by either paramedical staff, including nurses and lady health visitors or non-specialist physicians. Obstetricians provide care in referral hospitals.

*HDI:* 0.595 [8]

## Sub-Saharan Africa Sites (Figure 2)

**Figure 2:**
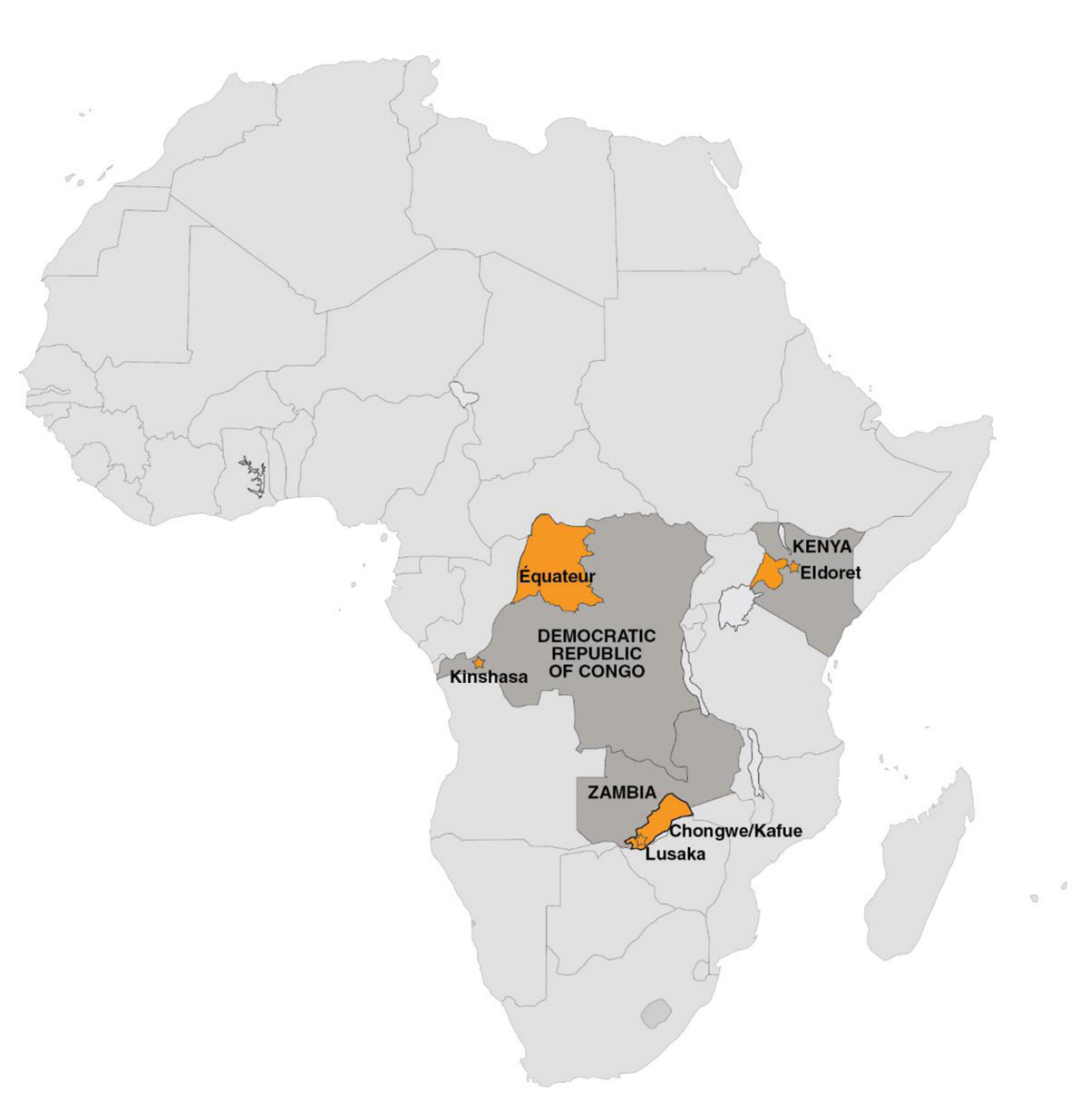
Sub-Saharan Africa Global Network Sites

### Kenya

*Location:* The research site is within the western region of Kenya in the counties of Busia, Bungoma and Kakamega, with the site coordinating center located in Eldoret.

*Setting:* There are 16 clusters served by 23 health facilities, most operated by the government and staffed by nurse-midwives and clinical officers and a single medical officer. Three hospitals in the area function as referral hospitals. Most physicians are generalists, with a small number of trained obstetricians and pediatricians.

*HDI*: 0.570 [7]

### Zambia

*Location*: The research site is within the districts of Kafue and Chongwe located south and east of the capital city of Lusaka, Zambia, where the site coordinating center is located.

*Setting*: There are ten clusters, eight of which have health posts. Care is provided primarily by nurse midwives in the health posts and by traditional birth attendants for home births. There are three district hospitals and a referral hospital in Lusaka. Specialty physicians are available in the referral hospital only.

*HDI:* 0.465 [9]

### Democratic Republic of Congo

*Location*: The research sites are in the northern province of Equateur, with the site coordinating center in Kinshasa.

*Setting*: There are 14 clusters, each served by a health center. Care in health centers is provided by nurses. There are three hospitals staffed by physicians, nurse midwives and nurses; no specialty physicians are available.

*HDI*: 0.338 (note: this is the HDI for the entire country; regional estimates were not available)[3].

## Latin American Sites (Figure 3)

**Figure 3:**
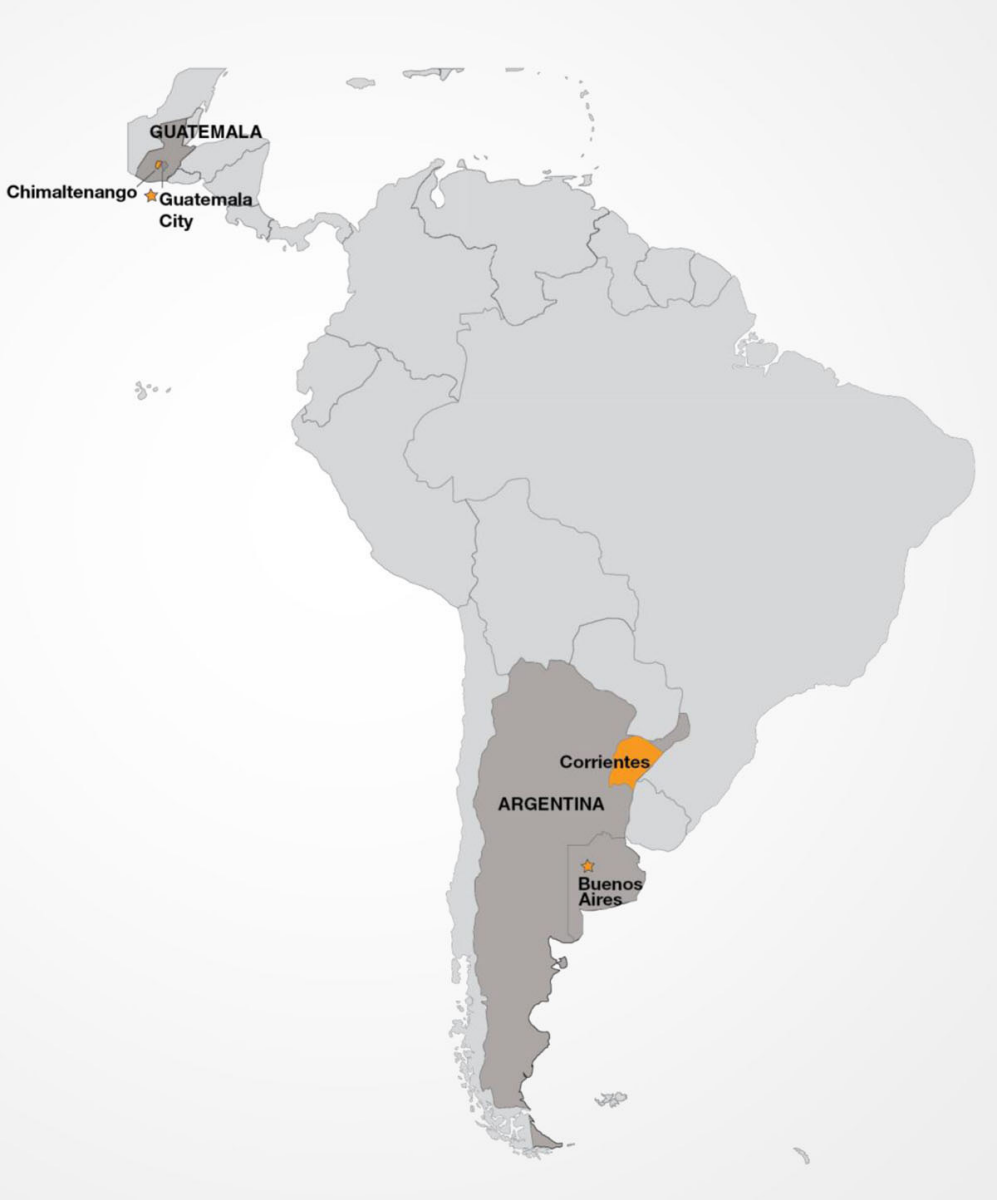
Latin American Global Network Sites

### Argentina

*Location:* The research site is within the provinces of Corrientes and Santiago del Estero are located in the northern region of Argentina, with the coordinating center in Buenos Aires.

*Setting:* There are six clusters, three in each province. Each cluster corresponds to a department (municipality) in which the vast majority of the births occur at a publicly-funded secondary level hospital. Care is provided primarily by physicians and midwives. There are two Provincial referral hospitals, each located in the provincial capital cities.

*HDI*: 0.828 [4]

### Guatemala

*Location:* The research site is within the Chimaltenango region is located in the Western Highlands of Guatemala, with the coordinating center in Guatemala City.

*Setting:* There are 17 clusters served by one referral hospital, 30 health centers, and 42 health posts. Maternal and infant care in the hospital is provided mainly by obstetricians and general physicians, in health centers by physicians and nurses, and in health posts by auxiliary nurses. *HDI:* 0.679 [5]

### MNHR Organization and Management

A MNHR Study Committee, constituted of investigators from each site and a representative from the NICHD and the Data Coordination Center (DCC; RTI, International), guides the general conduct of the MNHR (Appendix). The Committee oversees the use of MNHR data, data analyses and publications. Within each site, the MNHR is overseen by the senior foreign investigator and a country coordinator. In addition a field supervisor generally oversees the management of daily field activities for the MNHR. Each cluster employs a research administrator (RA) who is responsible for data collection and transfer to the DCC. Typically, the RAs are healthcare providers within the community. The RAs work closely with the existing healthcare service providers to help ensure that data describing pregnancies are comprehensive and accurate, as described elsewhere [10,11]. This work is facilitated by influential community leaders (e.g. village elders, birth attendants, and facility registries) [12]. In addition to field staff, each site employs a data manager who oversees data entry and data quality, including the process of resolving data edits.

## Methods

### Eligibility

Pregnant women are eligible for inclusion in the MNHR if they 1) reside or receive healthcare in the community (regardless of their site of delivery) or 2) deliver within the study cluster. Sites are encouraged to enroll women by 20 weeks of gestation; however, they can be enrolled at any time up to and including enrollment at the time of delivery. The overall goal is to capture the pregnancy outcomes of all eligible women. Each site uses site-specific strategies to achieve this goal. Birth attendants are sensitized to the study activities and are asked to report all deliveries in their communities, regardless of whether the delivery occurs at home or in a facility.

### Enrollment

Study staff maintain detailed maps of all health facilities and the location of birth attendants within each cluster (Figure 2). Staff routinely contact birth attendants active within clusters to document home deliveries, and review hospital and clinic logs for facility births on a monthly basis. The study team monitors cluster-level monthly data to identify trends over time (e.g. a declining or erratic birth rate) that might suggest failed enrollment of all women. At several sites, cell phone reporting by local village elders and traditional birth attendants has been effective [12]. Several sites have used annual household surveys to identify married women of reproductive age and to identify those likely to become pregnant in the ensuing year [10]. Where available, birth rates generated from the MNHR are compared to rates from local data sources such as ministry vital records to ensure accuracy in reporting.

### Data collection

The MNHR describes populations by collecting data describing demographic and health care characteristics and major outcomes of pregnancy. *A priori* definitions are used to classify certain outcomes and characteristics. For example, a stillbirth is defined using a modification of the World Health Organization (WHO) criteria: birth of a fetus weighing >500 g (or >22 weeks gestation) in which no signs of life (breathing, crying, heartbeat, or movement) were evident [13]. Fresh stillbirths have these signs but no signs of maceration. Gestational age is estimated using last menstrual period (LMP), or clinical data such as physical examination, ultrasound and other available information when LMP is unknown. The intent is to record measured birth weight taken within 48 hours of delivery using scales provided by the study. When birth weight is not obtained, weight is estimated with the goal of distinguishing infants weighing less than and greater than 1000g and 2500g. Models and pictures are used to teach these concepts to community birth attendants. Birth attendant is categorized as physician, nurse or equivalent, traditional birth attendant (TBA), family or unattended, and site of delivery is categorized as hospital, health center or home (including the TBA’s home or in-transit). Finally, antenatal care is defined as having at least one visit with a skilled health provider.

All definitions used by the MNHR are consistent with the WHO definitions, whenever possible. The primary reference guide for definitions is the *Integrated Management of Pregnancy and Childbirth: Pregnancy*, *Childbirth*, *Postpartum and Newborn Care: A guide for essential practice* [14].

Cause of deaths is assigned by the supervisory physicians based on their examination of all data available. Prior to 2014, there was no systematic method for assigning cause. This resulted in variability and inconsistency among sites in assignment of cause. For this reason, in 2014, a new system of assigning cause based on the collection of a defined data set and a computer algorithm was initiated [15].

Data are recorded at discrete time points, including demographic data at enrollment in the MNHR (which usually corresponds to the first antenatal care visit), and health care and outcomes at the time of delivery and at 42 days postpartum. Supplementary data are collected when a fetus, an infant or a mother dies at any time prior to 42 days postpartum.

Study staff collect all data on paper forms at each cluster; a supervisor performs a review of these forms for completeness and errors. Clerks enter these data into a computerized data management system, which contains basic logic checks. From each site, data are then transmitted to the DCC for central analyses, data edits, including inter- and intra-form consistency checks, and additional edits to ensure quality [16]. Data monitoring reports are reviewed monthly by each site team for completeness and consistency of data, as described elsewhere [11].

Annually, and in response to special inquiries, major outcomes of pregnancy and other perinatal health indicators are reported by the DCC. These include the following:

• Rates of pre-eclampsia/eclampsia, obstructed labor, hemorrhage, infection

• Site of delivery

• Birth attendant type

• Cesarean section rate

• Neonatal resuscitation rate

• Preterm birth rate

• Proportion of low birth weight newborns

• Maternal mortality rate

• Stillbirth rate

• Early neonatal mortality rate

• 28-day neonatal mortality rate

• Cause of maternal deaths within 42 days after delivery

• Cause of neonatal deaths within 28 days after delivery

## Training

The RAs receive training on the completion of data forms, schedule of data collection and process for editing data forms. Birth attendants are trained to collect data and assess basic clinical variables and outcomes, including differentiation of stillbirths from early neonatal deaths, birth weight and assessment of gestational age. Birth attendants are also taught to distinguish macerated from fresh stillbirths using pictures depicting forms of maceration.

RA’s have monthly meetings to review their data collection and refresher training on study definitions on an annual basis, with specific training held more frequently as needed.

## Quality assurance

Each site develops a monitoring plan to ensure the quality of the data. The monitoring plan has several components, including a timetable for responding to edits and an assessment of responsibility for completeness of data collection, data quality, data accuracy and data entry [11].

To assist with monitoring activities, the DCC prepares monthly monitoring reports of unexplained inconsistencies. They also prepare specially designed, site-specific programs to assist each site in monitoring. Finally, periodic site visits are conducted at each site by representatives of the NICHD, the DCC and the US investigator to review the completeness and quality of data collection.

## Ethical approval

The appropriate institutional review boards or ethics research committees of the participating institutions and the ministries of health of the respective countries approve the activities of the MNHR. Initially, approval was sought from the appropriate leader of the participating community. Informed consent for study participation is requested from each pregnant woman (and her partner when available). Study staff read an information page that has been translated into the local language to each women to inform her of the study purpose. There are no monetary reimbursements to participants, and there is no remuneration to the communities participating in the study. The Global Network Data Monitoring Committee, appointed by the NICHD, oversees and reviews activities of the MNHR at annual meetings.

## MNHR Population Characteristics 2010-2013

Since the inception of the MNHR in 2008, more than 450,000 pregnant women have been enrolled. During the calendar years 2010-2013, 283,496 pregnant women were enrolled in the MNHR, representing 98.8% of those eligible (Table 2). Rates of enrollment by site varied from 95.0% in Argentina to 99.9% in the two Indian sites (data not shown). Of the pregnancies registered, we were able to obtain delivery data for 98.8% of women and 42-day follow-up data for 98.4% of those enrolled. No site had >5% loss to follow-up. The time of registration varied by site. For example 96% of women were registered within 4 weeks of delivery in Argentina. By contrast, in four sites, Belgaum, Nagpur, Pakistan and Zambia, over half of women were enrolled prior to the third trimester. Recently, efforts have been made to enroll women earlier in pregnancy so that early outcomes (e.g. miscarriage) can be counted with accuracy.

**Table 2:** Demographic Features of Women Enrolled in the Maternal Newborn Health Registry 2010-2013

	Argentina	Guatemala	Zambia	Kenya	Pakistan	Nagpur	Belgaum	Total
Pregnant women, N	9,944	30,806	28,176	36,509	51,794	41,678	84,589	283,496

Maternal age, N (%)								

< 20	2,684 (27.1)	5,067 (16.5)	7,116 (25.3)	7,869 (21.9)	2,018 (3.9)	833 (2.0)	8,134 (9.6)	33,721 (11.9)

20-35	6,447 (65.2)	22,467 (73.0)	18,757 (66.8)	26,545 (73.9)	46,682 (90.5)	40,693 (97.7)	76,214 (90.2)	237,805 (84.2)

> 35	760 (7.7)	3,252 (10.6)	2,221 (7.9)	1,524 (4.2)	2,905 (5.6)	120 (0.3)	163 (0.2)	10,945 (3.9)

Education, N (%)								

No formal education	254 (2.6)	5,983 (19.4)	2,922 (10.5)	1,110 (3.1)	42,879 (83.1)	1,269 (3.0)	17,224 (20.5)	71,641 (25.4)

Primary	6,146 (62.7)	19,436 (63.1)	15,412 (55.1)	25,637 (71.3)	3,908 (7.6)	7,233 (17.4)	27,884 (33.2)	105,656 (37.5)

Secondary	3,258 (33.2)	5,037 (16.4)	9,123 (32.6)	7,897 (22.0)	3,028 (5.9)	24,761 (59.5)	31,101 (37.0)	84,205 (29.9)

University+	152 (1.5)	333 (1.1)	500 (1.8)	1,303 (3.6)	1,767 (3.4)	8,363 (20.1)	7,745 (9.2)	20,163 (7.2)

Parity, N (%)								

0	3,232 (32.8)	8,618 (28.0)	7,609 (27.1)	9,046 (25.2)	10,749 (20.8)	19,941 (47.9)	35,742 (42.5)	94,937 (33.7)

1-2	3,831 (38.9)	11,010 (35.7)	10,554 (37.5)	13,881 (38.6)	16,563 (32.1)	20,623 (49.5)	42,672 (50.8)	119,134 (42.2)

> 2	2,794 (28.3)	11,171 (36.3)	9,944 (35.4)	13,020 (36.2)	24,341 (47.1)	1,103 (2.6)	5,657 (6.7)	68,030 (24.1)

Mean (std)	1.9 (2.2)	2.4 (2.6)	2.1 (2.0)	2.2 (2.1)	3.0 (2.8)	0.7 (0.8)	0.9 (1.0)	1.7 (2.1)

Table 2 lists characteristics of women enrolled in the MNHR at each site from 2010 to 2013. These data highlight differences in demography among sites. For example, women tended to be older in the South Asian sites compared to other sites, and were more likely to be primiparous at the Indian sites. Over 80% of women at the Pakistani site had no formal education. Table 3 lists selected features of perinatal care of the MNHR cohort. The most striking differences among sites were the percent of women delivered by cesarean section, attendance at delivery by a physician and delivery in a facility. The associations of demographic and perinatal care characteristics with pregnancy outcomes are explored in other manuscripts in this supplement [10,11,18-29].

**Table 3:** Characteristics of Deliveries of Women in the Maternal Newborn Health Registry 2010-2013

	Argentina	Guatemala	Zambia	Kenya	Pakistan	Nagpur	Belgaum	Total
Deliveries, N	9,902	30,262	27,877	35,667	50,275	41,424	84,581	279,988

At least one ANC visit, N (%)								

Yes	9,312 (95.0)	29,696 (98.2)	27,547 (98.9)	34,643 (97.2)	41,792 (83.5)	41,361 (99.9)	84,145 (99.7)	268,496 (96.1)

No	494 (5.0)	533 (1.8)	307 (1.1)	1,003 (2.8)	8,243 (16.5)	30 (0.1)	218 (0.3)	10,828 (3.9)

Delivery mode, N (%)								

Cesarean	3,469 (35.0)	5,580 (18.4)	305 (1.1)	550 (1.5)	4,489 (9.4)	7,701 (19.6)	11,211 (14.0)	33,305 (12.3)

Vaginal/Vaginal assisted	6,430 (65.0)	24,673 (81.6)	27,455 (98.9)	35,112 (98.5)	43,420 (90.6)	31,506 (80.4)	68,583 (86.0)	237,179 (87.7)

Birth attendant, N (%)								

Physician	7,163 (72.4)	12,951 (42.8)	692 (2.5)	717 (2.0)	12,687 (25.3)	24,383 (58.9)	48,871 (57.8)	107,464 (38.4)

Nurse/Midwife/HW	2,678 (27.1)	543 (1.8)	15,433 (55.4)	14,505 (40.7)	13,080 (26.1)	14,223 (34.4)	28,307 (33.5)	88,769 (31.7)

TBA	2 (0.0)	16,675 (55.1)	7,093 (25.4)	16,038 (45.0)	21,961 (43.7)	1,178 (2.8)	2,002 (2.4)	64,949 (23.2)

Family/Other	51 (0.5)	92 (0.3)	4,656 (16.7)	4,407 (12.4)	2,475 (4.9)	1,616 (3.9)	5,377 (6.4)	18,674 (6.7)

Delivery location, N (%)								

Hospital	9,789 (98.9)	12,048 (39.8)	3,453 (12.4)	4,616 (12.9)	14,186 (28.3)	27,433 (66.3)	55,761 (66.0)	127,286 (45.5)

Clinic	15 (0.2)	1,367 (4.5)	13,482 (48.4)	10,245 (28.7)	12,296 (24.5)	10,933 (26.4)	20,777 (24.6)	69,115 (24.7)

Home/Other	93 (0.9)	16,846 (55.7)	10,939 (39.2)	20,805 (58.3)	23,730 (47.3)	3,031 (7.3)	7,976 (9.4)	83,420 (29.8)

Gender, N (%)								

Male	5,153 (51.8)	15,494 (50.9)	14,703 (52.8)	18,300 (50.7)	25,564 (52.7)	20,555 (52.3)	41,663 (52.0)	141,432 (51.9)

Female	4,797 (48.2)	14,948 (49.1)	13,142 (47.2)	17,796 (49.3)	22,975 (47.3)	18,763 (47.7)	38,506 (48.0)	130,927 (48.1)

Birth weight (measured)								

< 2500g	646 (6.5)	3,927 (13.0)	1,494 (5.4)	1,061 (3.0)	6,657 (14.7)	6,300 (16.1)	11,557 (14.5)	31,642 (11.9)

≥ 2500g	9,302 (93.5)	26,361 (87.0)	25,987 (94.6)	34,257 (97.0)	38,507 (85.3)	32,800 (83.9)	67,892 (85.5)	235,106 (88.1)

Mean (std)	3286.1 (578.2)	2983.2 (469.1)	3119.0 (473.4)	3231.5 (486.7)	2915.1 (551.6)	2662.2 (418.8)	2746.4 (439.9)	2912.3 (517.5)

TBA=traditional birth attendant

## Strengths and Limitations

The Global Network MNHR provides prospectively collected, population-based pregnancy outcome for defined geographic regions within low- and middle-income countries. One of its strengths is the representative nature of the MNHR, which reduces bias of facility-based registries. Additionally, because it is a prospective study, it reduces some of the recall bias associated with periodic surveys [30,31]. Additionally, the MNHR uses standardized definitions and methods across disparate sites which allows for comparison. Finally, the large enrollment (approximately 70,000 annual pregnancies) allows for precision in documentation of relatively rare events, such as maternal mortality.

One of the limitations of the MNHR is the difficulty in ensuring the inclusion of all pregnancies, and especially those with early pregnancy loss. We acknowledge that pregnancies resulting in miscarriages and terminations are currently under-reported. Historically, at most sites, enrollment in the MNHR has been at the time of the first prenatal visit, and this was often after the first trimester. Some sites are now using strategies to encourage earlier initiation of prenatal care. It is hoped that this will result in earlier registration. There is also the potential for non-enrollment of pregnancies that are managed outside of the health care system. Despite this potential loss, we believe that our enrolled populations approach 100% of all women whose pregnancies reach the second trimester, based on reviews at each site with other existing data. For example, in a recent report from the Belgaum site, birth rates reported by the MNHR were higher than projected based on ministry data and other sources, indicating that the surveillance for the MNHR was more comprehensive than the available census data [10]. Some sites encounter challenges in tracking the outcomes of pregnant women who migrate in or out of the study clusters, for example women who travel to the homes of their mothers at the time of delivery. To address these challenges, numerous systems have been developed and, through monitoring, a relatively stable enrollment rate has been achieved at the affected study clusters [11].

Some challenges exist in categorizing critical pregnancy outcomes. For example, when birth occurs at home unattended by a skilled birth attendant, the proper classification of intrapartum stillbirth versus very early neonatal death [14] and macerated versus non-macerated stillbirth are particularly challenging [15]. Finally, determining accurate birth weights of certain groups of infants is difficult. The weight of stillbirths is often not possible to obtain because weighing a dead infant is not culturally acceptable in many communities. Acquiring an accurate birth weight of a live-born infant delivered at home is often difficult because the infant cannot be weighed within a sufficiently brief period after birth. To overcome this challenge, strategies have included home visitation and providing village chiefs with scales [12].

## Discussion and Conclusions

Developing public policy and improving public health in countries with poor perinatal outcomes is, in part, dependent upon understanding the outcome of every pregnancy. Because the worst pregnancy outcomes typically occur in countries with limited health registration systems and vital records, alternative registration systems may prove to be highly valuable in providing data. One alternative is a survey system, such as the Demographic and Health Survey (DHS). The DHS has conducted surveys in more than 90 low- and middle-income countries since 1984 [30]. It is widely used for country comparisons but is handicapped in this capacity because it is often adapted by individual countries to suit national needs for specific data [31]. By contrast, the MNHR has the advantage of using the same data set, data gathering techniques and standard definitions across all sites. The MNHR also is an ideal tool for evaluating the effectiveness of strategies of care because, unlike with the use of periodic surveys, data is collected continuously over time within the same population-based cohort. This enables investigators to determine the impact of interventions to improve outcomes, to monitor trends over time, and to evaluate the changing patterns of perinatal care to inform health policy.

In this supplement, there are a series of manuscripts that highlight the utility of the MNHR [10,11,18-27]. They include manuscripts that describe processes of care that define the quality of antenatal care and major pregnancy outcomes, both among all women enrolled in the MNHR and in selected groups (e.g. adolescent mothers). Finally, problems and outcomes unique to single sites are explored.

## List of Abbreviations

NICHD: *Eunice Kennedy Shriver* National Institute of Child Health and Human Development; MNHR: Global Network Maternal Newborn Health Registry; HDI: Human Development Index; DCC: Data Coordination Center; RA: research administrator traditional birth attendant; LMP: last menstrual period; WHO: World Health Organization; TBA: traditional birth attendant; DHS: Demographic and Health Survey

## Competing Interests

The authors have no competing interests.

## Authors Contributions

CLB developed the content outline and first draft of the manuscript.

MB developed sections of the manuscript and edited its content.

EMM assisted with reporting of data and edited the content.

RLG, SSG, OP and WAC edited the content. AG edited the content and provided country-specific HDIs.

JM provided data for tables and other facts describing sites.

MK-T and MM edited the content.

All authors reviewed and approved the final manuscript.

## Site coordination teams

Argentina: Fernando Althabe, José M Belizán, Agustina Mazzoni, Maria Luisa Cafferata, Mabel Berrueta, (Institute for Clinical Effectiveness and Health Policy), A Ciganda (Clinical and Epidemiological Research Unit Montevideo), Pierre M. Buekens (Tulane School of Public Health and Tropical Medicine); Democratic Republic of Congo: Antoinette Tshefu, Adrien Lokangako, (Kinshasa School of Public Health); Melissa Bauserman, Carl L Bose (UNC Chapel Hill); Guatemala: Ana Garces, Lester Figueroa (Fundación para la Alimentación y Nutrición de Centro América y Panamá), K. Michael Hambidge, Nancy F. Krebs (University of Colorado); India (Belgaum): Bhalchandra Kodkany, Shivaprasad S Goudar, Sangappa M Dhaded, Narayan V Honnungar, Manjunath S Somannavar, Sunil S Vernekar, Shivanand C Mastiholi, Amit P Revankar (KLE University’s Jawaharlal Nehru Medical College, Belgaum); Ashalata M Mallapur, Umesh V Ramadurg, Geetanjali M Katageri (S Nijalingappa Medical College, Bagalkot); Frances J. Jaeger, Richard J. Derman (Christiana Care Health Services, Delaware); India (Nagpur): Archana Patel (Indira Gandhi Government Medical College; Lata Medical Research Foundation), P. S. Kalsait, Kunal Kurhe, Patricia L. Hibberd (Massachusetts General Hospital); Kenya: Fabian Esamai, Irene Marete; Constance Tenge, Paul Nyongesa, Silas Ayunga (Moi University School of Medicine), Edward A. Liechty, Sherri Bucher (Indiana University); Pakistan: Omrana Pasha, Sarah Saleem Farnaz Naqvi, Shiyam Sunder, Zaheer Habib (Aga Khan University), Neelofar Sami (Willows Foundation), Margo Harrison, Robert L. Goldenberg (Columbia University); Zambia: Elwyn Chomba; Musaku Mwenche (University of Zambia); Melody Chiwila (CIDRZ), Waldemar A. Carlo (University of Alabama at Birmingham); Data Coordinating Center: Elizabeth M. McClure, Janet L. Moore Norman Goco, Stephen D. Litavecz, Dennis D. Wallace (RTI International); *Eunice Kennedy Shriver* National Institute of Child Health and Human Development: Marion Koso-Thomas, Menachem Miodovnik; Global Network Chair: Alan H. Jobe (University of Cincinnati Children’s Hospital).

## Site field teams

Argentina: Ana Maria Dominguez; Tania Lima Pérez; María José Pellegrini; Cristina Ganduglia; Rita Agüero; Diego Espinosa; Lucas Urquiza; Andrea Barea; Daniela Moreli; Belgaum, India: Ganapti V Lakkundi; Harsha S Patil; SM Chowdappanavar; Sanjay S Siddannavar; V N Kpasi; Satish P Malali; Ramesh Haller; Jagadish K Jingi; RS Balikaj; MB Rayar; Vinod D Gasti; Mahesh J Kumbar; Iranna I Kalli; Bhujabali D Yalagudri; N A Gotyal; Chandrakanth Sutar; Sukanya Handage; Anil Salagare; Ashok S Kumbar; Salim A Mujawar; Laxmi B Herawade; B N PatIl; Sheetal Ingle; Ravasab Sankaje; Uday S Kudachi; Shahid Gadekai; Shivanand Mulakuri; Adivesh Munavalli; Dayanand Shirur; Rajendra K Kilabanur; Sanjay S Doddamani; Santosh G Shettar; S V Melavanki; Shridhar S Pattar; Kusuma Y Magi; Vikas B Parwatikar; P M Itagi; K F Mayachari; Venkanna Emmi; Basavraj S Madali; Vinayak Mhetre; Nagraj Khade; Tanaji Khade; Sachin Mastiholi; G S Kengapur; B B Avoji; Democratic Republic of Congo: Charles Kombi, Michel Kalonji; Kenya: Carolyne Chemweno; Guatemala: Evelyn Morales, Marta Lidia Aguilar; Nagpur, India: Jayant Shamkuwar, Ravi Petkar, Shashank Ambhore, Atul Chopde, Nitesh Nikose, Atul Andelkar; Pakistan: Zahid Soomro; Amirzadi Khaskheli; Irfan Karim; Hussein Shaikh; Asma Amir; Samreen Gul; Rabia Samejo; Fahmida Gul; Zambia: Royce Shamapu; Rosemary Sotelli; Jessica Kapesa; Emmanuel Lwao; Lasco Ntanisha; Edwin Cheelo; Mavis Liteta; Raymond Ngwenya; Martha Katenga; Jean Mwanza; Shira Makwama ; Denis Kapamulomo ; Doreen Kadipa ; Carol Mubila ; Love Kasakula ; Sara Mukula ; Eness Sappi ; Mwazi Sikalenge ; Salome Lyanoonga ; Emilliy Muchimba

The study was funded by grants (U01HD040636, U10HD078437, U10HD076461, U10HD076465, U10HD076457, U10HD078439, U10HD078438, and U10HD076474) from the *Eunice Kennedy Shriver* National Institute of Child Health and Human Development of the US National Institutes of Health.

## Peer review

Reviewer reports for this article can be found in Additional file 1.

## Supplementary Material

Additional file 1 Click here for file

## Declarations

This article has been published as part of *Reproductive Health* Volume 12 Supplement 2, 2015: Research reports from the NICHD Global Network for Women’s and Children’s Health Research Maternal and Newborn Health Registry. The full contents of the supplement are available online at http://www.reproductive-health-journal.com/supplements/12/S2. Publication of this supplement was supported by grants from the Eunice Kennedy Shriver National Institute of Child Health and Human Development to RTI International.

## References

[B1] Carlo WA Goudar SS Jehan I Chomba E Tshefu A Garces A Newborn-care training and perinatal mortality in developing countries N Engl J Med 2010 362 7 614 23 doi:362/7/614 [pii]10.1056/NEJMsa0806033 10.1056/NEJMsa0806033 20164485 PMC3565382

[B2] Goudar SS Carlo WA McClure EM Pasha O Patel A Esamai F The Maternal and Newborn Health Registry Study of the Global Network for Women's and Children's Health Research Int J Gynaecol Obstet 2012 118 3 190 3 doi:10.1016/j.ijgo.2012.04.022 10.1016/j.ijgo.2012.04.022 22738806 PMC3417109

[B3] Human Development Index and its components http://hdr.undp.org/en/content/table-1-human-development-index-and-its-components

[B4] Argentina en un mundo incierto Asegurar el desarrollo humano en el sigle XXI http://www.ar.undp.org/content/dam/argentina/Publications/Desarrollo Humano/ARGentina-PNUD-INDH 2013.pdf

[B5] Cifras para el desarrollo humano Chimeltenango http://desarrollohumano.org.gt/sites/default/files/04 Fasciculo Chimaltenango.pdf

[B6] Inequality-adjusted Human Development Index for India's States 2011 http://www.undp.org/content/dam/india/docs/inequality_adjusted_human_development_index_for_indias_state1.pdf

[B7] Kenya Institute for Public Policy Research and Analysis http://www.kippra.org/downloads/Kenya Economic Report 2013.pdf

[B8] United Nations Development Programme in Pakistan http://www.pk.undp.org

[B9] Zambia Human Development Report 2011 http://planipolis.iiep.unesco.org/upload/Zambia/Zambia_NHDR_2011_en.pdf

[B10] Kodkany B Derman RJ Honnungar N Tyagi N Goudar SS Mastiholi S Establishment of a Maternal Newborn Health Registry in the Belgaum District of Karnataka, India 10.1186/1742-4755-12-S2-S3 PMC4464217 26062791

[B11] Goudar SS Stolka KB Koso-Thomas M McClure EM Carlo WA Goldenberg RL The Global Network’s Maternal Newborn Health Registry: Data quality monitoring and performance metrics.

[B12] Gisore P Shipala E Otieno K Rono B Marete I Tenge C Community based weighing of newborns and use of mobile phones by village elders in rural settings in Kenya: a decentralised approach to health care provision BMC Pregnancy Childbirth 2012 12 15 doi:10.1186/1471-2393-12-15 10.1186/1471-2393-12-15 22429731 PMC3344691

[B13] Pregnancy, Childbirth, Postpartum and Newborn Care: A guide for essential practice 2006 Geneva: World Health Organization 26561684

[B14] Integrated Management of Pregnancy and Childbirth: Pregnancy, Childbirth, Postpartum and Newborn Care: A guide for essential practice 2007 Geneva: World Health Organization 26561684

[B15] McClure EM Bose CL Garces A Esamai F Goudar SS Patel A Global Network for Women’s and Children’s Health Research: A System for Low-Resource Areas to Determine Probable Causes of Stillbirth, Neonatal, and Maternal Death 10.1186/s40748-015-0012-7 PMC4823684 27057328

[B16] Goldenberg RL McClure EM Jobe AH Kamath-Rayne BD Gravet MG Rubens CE Stillbirths and neonatal mortality as outcomes Int J Gynaecol Obstet 2013 123 3 252 3 10.1016/j.ijgo.2013.06.020 24050480 PMC4349406

[B17] Gold KJ Abdul-Mumin AR Boggs ME Opare-Addo HS Lieberman RW Assessment of "fresh" versus "macerated" as accurate markers of time since intrauterine fetal demise in low-income countries Int J Gynaecol Obstet 2014 125 3 223 7 10.1016/j.ijgo.2013.12.006 24680841 PMC4025909

[B18] Bauserman M Lokangaka A Thorsten V Tshefu A Goudar SS Esamai F Risk factors for maternal death in low- and middle-income countries: a prospective longitudinal cohort analysis 10.1186/1742-4755-12-S2-S5 PMC4464034 26062992

[B19] McClure EM Saleem S Goudar SS Moore JL Esamai F Garces A Stillbirth trends in low-middle income countries 2010 - 2013: A population-based, multi-country cohort study from the Global Network 10.1186/1742-4755-12-S2-S7 PMC4464024 26063292

[B20] Dhaded SM Somannavar M Vernekar S Goudar SS Ramadurg U Mwenche M Neonatal mortality and risk factors 2010 - 2013: A prospective, population-based Global Network cohort study

[B21] Bucher S Marete I Tenge C Liechty EA Esamai F Patel A A prospective, observational study of antenatal care attendance and coverage of selected interventions in Argentina, Guatemala, India, Kenya, Pakistan and Zambia 10.1186/1742-4755-12-S2-S12 PMC4464209 26063483

[B22] Harrison MS Ali S Pasha O Saleem S Althabe F Berrueta M A prospective study of maternal, fetal, and neonatal outcomes in the setting of prolonged labor, obstructed labor and failure to progress in low- and middle-income countries 10.1186/1742-4755-12-S2-S9 PMC4464213 26063492

[B23] Pasha O Saleem S Ali S Goudar SS Garces A Esamai F Diverging maternal, fetal and neonatal outcomes: Pakistan and other low and middle income countries in the Global Network’s Maternal Newborn Health Registry

[B24] Garces A Mcclure EM Hambidge KM Krebs NF Figueroa L Aguilar ML Trends in perinatal deaths from 2010 to 2013 in the Guatemalan Western Highlands 10.1186/1742-4755-12-S2-S14 PMC4464607 26062407

[B25] Goudar SS Goco N Somannavar MS Vernekar SS Mallapur AA Moore JL Institutional deliveries and perinatal and neonatal mortality in Southern and Central India 10.1186/1742-4755-12-S2-S13 PMC4464025 26063586

[B26] Patel A Bucher S Pusedekar Y Esamai F Krebs NF Goudar SS Lack of early initiation of breast feeding is associated with late newborn and early infant death among rural populations in low and middle income countries: A prospective, cohort study

[B27] Marete I Tenge C Chemweno C Bucher S Pasha O Goudar SS Loss to followup among pregnant women in a multi-country, community-based maternal and newborn health registry: A prospective, cohort study 10.1186/1742-4755-12-S2-S4 PMC4464022 26062899

[B28] Althabe F Moore J Gibbons L Berrueta M Goudar SS Chomba E Adverse maternal and perinatal outcomes in adolescent pregnancies: The Global Network’s Maternal Newborn Health Registry study 10.1186/1742-4755-12-S2-S8 PMC4464033 26063350

[B29] Pasha O Goudar SS Patel A Garces A Esamai F Chomba E Postpartum Contraceptive Use and Unmet Need for Family Planning in 5 low-income countries Reproductive Health in press 10.1186/1742-4755-12-S2-S11 PMC4464604 26063346

[B30] Measure DHS 2013 http://www.measuredhs.com/Measure

[B31] Footman K Benova C Goodman C Macleod D Lynch CA Penn-Kekana L Campbell OMR Using multi-country household surveys to understand who provides reproductive and maternal health services in low- and middle-income contries: a critical appraisal of the Demographic and Health Surveys Trop Med Intern Health 2015 20 5 589 606 10.1111/tmi.12471 PMC4409817 25641212

