# Supplementary material for: The Global Network Maternal Newborn Health Registry: a multi-national, community-based registry of pregnancy outcomes
Source: Reprod Health. 2015 Jun 8;12(Suppl 2):S1. doi: 10.1186/1742-4755-12-S2-S1 (PMC4464212; doi:10.1186/1742-4755-12-S2-S1)
Supplement: Additional file 1 [file 1742-4755-12-S2-S1-S1.pdf]

Referee's comments to the authors– this sheet WILL be seen by the author(s) and published with the article

Title	The Global Network Maternal Newborn Health Registry: a multi-national, community-based registry of pregnancy outcomes
Author(s)	Carl L Bose, Melissa Bauserman, Robert L Goldernberg, Shrivaprasad S Goudar, Elizabeth M McClure, Omrana Pasha, Waldermar A Carlo, Ana Garces, Janet L Moore, Menachem Miocovnik, Marion Koso-Thomas
Referee's name	Eve Lackritz

When assessing the work, please consider the following points, where applicable:

1. Is the question posed by the authors new and well defined?
2. Are the methods appropriate and well described, and are sufficient details provided to replicate the work?
3. Are the data sound and well controlled?
4. Does the manuscript adhere to the relevant standards for reporting and data deposition?
5. Are the discussion and conclusions well balanced and adequately supported by the data?
6. Do the title and abstract accurately convey what has been found?
7. Is the writing acceptable?

Please make your report as constructive and detailed as possible in your comments so that authors have the opportunity to overcome any serious deficiencies that you find and please also divide your comments into the following categories:

- Major Compulsory Revisions (which the author must respond to before a decision on publication can be reached)
- Minor Essential Revisions (such as missing labels on figures, or the wrong use of a term, which the author rin
- Discretionary Revisions (which are recommendations for improvement but which the author can choose to ignore)

Where possible please supply references to substantiate your comments.

When referring to the manuscript please provide specific page and paragraph citations where appropriate.

General comments: The manuscript outlines the Global Network Maternal Newborn Health Registry that was developed as a platform to assess maternal and neonatal outcomes used for clinical trials in areas that lack vital statistics. A brief description of the GN sites is included. Major outcomes of interest are listed, including rates of major maternal complications, preterm birth, stillbirth, maternal mortality, etc.

The stated objectives of the registry are to provide data for public health policy, improving public health, and to track trends over time. The manuscript provides a general description of each site and what was measured. No summary data or methodological assessments are provided that examine validation of measurements of primary outcomes or confidence intervals around those estimates. Without that, it is unclear whether or not the data represent accurate estimates of rates or have sufficiently narrow confidence intervals to track trends. No data are provided on sample size of each site or of primary outcomes. Beyond a platform for clinical trials, the authors do not present data on how this project added more or higher quality

(

data of primary outcomes compared with other population-based estimates provided by DSS, DHS, MICS, and other methods. Although numbers are not reported, it is unlikely that this registry would serve as a representative method for some of the key outcomes, for example, maternal mortality. Information on assessment of quality of cause of death data is not reported, or how results of these cause of death data compared with other methods and estimates.

An important publication from the Global Network reported newborn resuscitation decreased stillbirth but not neonatal mortality rates. Another published study of antenatal corticosteroids for treatment of preterm birth used birthweight as a surrogate for gestational age. As such, it appears that accurate measurement of the primary outcomes of the project, including stillbirth and preterm birth, were likely not accurately measured. Under Limitations, the manuscript states that, “The proper classification of…stillbirth versus very early neonatal death is a particular challenge.” “Attempts are made to enroll women by 20 weeks of gestation” (p. 6), but data are not reported on when women were enrolled (e.g. median, range). Rather than report claims, the manuscript would benefit from quantitative assessments of the registry data and what methods were employed to validate results. No data are presented for the reader to assess the quality, accuracy, and completeness of the data reported. The paper would benefit of a more cautious use of terms such as “precise,” “high quality,” and “accurate” to describe the registry data without data to support these claims.

Major compulsory revisions:

Rather than a descriptive report of the system, the manuscript would make a more meaningful contribution to the literature if it included a quantitative report of the system, including data on the sample size, estimates of primary outcomes measured at each site, and confidence intervals around those estimates (perhaps in a table). Instead of claims of data quality, an analytic comparison of the registries with other methods used to estimate primary outcomes would be useful. The Discussion could include results of validation and comparisons with other population-based estimates of medical complications of pregnancy and birth outcomes.

Minor essential revisions:

Discretionary revisions:

Referee's comments to the authors– this sheet WILL be seen by the author(s) and published with the article

Title	The Global Network Maternal Newborn Health Registry: a multi-national, community-based registry of pregnancy outcomes
Author(s)	Carl L Bose, Melissa Bauserman, Robert L Goldernberg, Shrivaprasad S Goudar, Elizabeth M McClure, Omrana Pasha, Waldermar A Carlo, Ana Garces, Janet L Moore, Menachem Miocovnik, Marion Koso-Thomas
Referee's name	Susannah Leisher

When assessing the work, please consider the following points, where applicable:

1. Is the question posed by the authors new and well defined?
2. Are the methods appropriate and well described, and are sufficient details provided to replicate the work?
3. Are the data sound and well controlled?
4. Does the manuscript adhere to the relevant standards for reporting and data deposition?
5. Are the discussion and conclusions well balanced and adequately supported by the data?
6. Do the title and abstract accurately convey what has been found?
7. Is the writing acceptable?

Please make your report as constructive and detailed as possible in your comments so that authors have the opportunity to overcome any serious deficiencies that you find and please also divide your comments into the following categories:

- Major Compulsory Revisions (which the author must respond to before a decision on publication can be reached)
- Minor Essential Revisions (such as missing labels on figures, or the wrong use of a term, which the author can be trusted to correct)
- Discretionary Revisions (which are recommendations for improvement but which the author can choose to ignore)

Where possible please supply references to substantiate your comments.

When referring to the manuscript please provide specific page and paragraph citations where appropriate.

General comments:

The Global Network for Women's and Children's Health Research was established to promote women's and children's health in "resource-limited countries". An important element of the Global Network is the Maternal Newborn Health Registry (MNHR), which aims to collect data on all pregnancy outcomes in all Global Network sites, for use in Global Network clinical trials. This paper briefly describes the MNHR methodology and coverage results for the period 2010 to 2013.

The MNHR is an important program for both the data it provides and the model it offers. This paper is a necessary reference for other papers in this supplement, as well as being a helpful update to prior papers on MNHR coverage, and a potentially useful resource for researchers in low-resource countries interested in collaboration with the Global Network for their own women's and children's health objectives. Previously published papers have provided 2010-2012 coverage results and information on MNHR methodology. This paper adds the latest coverage data from 2013 and provides additional details on methodology. It will be a useful addition to the literature.

Major compulsory revisions:

None**.**

Minor essential revisions:

Overall:

1. The paper should follow the format required by the journal. The Introduction should be renamed “Background”; all the sections from “MNHR Organization and Management” through “Ethical approval” should be contained within a single section called “Methods” (including the section that is already named “Methods”); the section titled “Enrollment in the MNHR 2010-2013” should be renamed “Results”; and the sections “Limitations” and “Conclusions” should both be within a single section “Discussion and Conclusions”. Alternatively, Results and Discussion/Conclusions could be combined into a single section as they are quite short. This will not only ensure alignment with requirements but will also assist the reader.

Title:

2. To ensure alignment with author guidelines, the title should include the study design. To make it easier for other researchers to find this paper in a literature search, I suggest the title be further revised to include the countries of the Global Network sites and the years for which data are presented, eg “The Global Network’s Maternal Newborn Health Registry: A prospective community-based pregnancy registry in Argentina, Guatemala, India, Kenya, Pakistan, and Zambia, 2010-2013”.

Abstract:

3. The Abstract should make clear the main objective of the paper, which is to provide updated coverage data and methodology details as a reference for other papers in the supplement.

Background (“Introduction”):

4. (parag. 1) The Background should restate the main objective of the paper in line with point 3 above.
5. (parag. 4) The sentence discussing data collection should reference the supplement paper on data quality monitoring.
6. Prior publication of MNHR coverage data should be specified (for instance, Saleem 2014 [Saleem, S., et al. (2014). "A prospective study of maternal, fetal and neonatal deaths in low- and middle-income countries." Bull World Health Organ 92(8): 605-612] gives coverage data for 2010-2012 and Goudar (already referenced) for 2010), with clarification that the present paper provides updated data through 2013.

Methods (multiple sections):

7. The parag on Quality Assurance should refer to the separate paper on this topic in the supplement.
8. A brief sentence should clearly list the major pregnancy outcomes collected (stillbirth, maternal mortality, early and late neonatal mortality…) and distinguish these from the other variables that are collected.
9. Any exclusion criteria should be described briefly in the parag that details eligibility criteria.
10. To be in full alignment with journal requirements for this section, a sentence clearly stating the study design should be included.
11. Mention should be made of how multiple births to the same woman were addressed (non-independence of data), or reference made to papers in the supplement that explain this.
12. The names of any systems used for classifying causes of death should be provided in the parag listing definitions of variables used.
13. Although this is a population-based registry with nearly 100% coverage, study power should still be mentioned. At minimum, (if the case), it should be stated that power calculations are included in all the relevant papers in the supplement. Moreover, although the nature of the registry (as a source of data for studies on a range of outcomes and sub-populations) means it is not possible to make any comprehensive statements on power, still, to increase the utility of this paper for prospective researchers, a brief statement of the sizes of effect the study is powered to detect would be useful. For example, the statement could address just the key MNHR outcomes (stillbirth, neonatal mortality, and maternal mortality) in each study site (eg, “In the xx study site, the annual sample size of approximately xx is sufficient to detect a reduction of xx% in maternal mortality with xx% power”).
14. It is stated that women are eligible for inclusion not only if they reside in the cluster but also if they deliver in the cluster. Are women who live outside the cluster returning to their maternal homes within the cluster to deliver? If so, an explanation should be added of how the population size from which enrolment rates are calculated is determined.

Results (“Enrollment in the MNHR 2010-2013”):

15. At the end of the sentence on loss to follow-up, reference should be made to the relevant paper on this topic in the supplement.
16. Any notable changes in rates of enrollment and coverage of pregnancy outcomes at birth and day 42 over the four-year period (per site) should be mentioned, as this would provide useful context for the reader.
17. Very high coverage notwithstanding, the main reasons for non-participation at enrollment and loss to follow-up at birth and Day 42 should be briefly mentioned, as per STROBE checklist for cohort studies.
18. Reference should be made to other papers in the supplement that will provide the basic demographic data and key outcome measures for participants.

Discussion and conclusions ("Limitations" and "Conclusion"):

19. For the limitations discussed (difficulty of including all pregnancies; potential misclassification of intrapartum stillbirth/early neonatal death and of macerated/fresh stillbirth; and possible measurement error in and other challenges of obtaining birthweight), the potential implications of each type of limitation for results should be mentioned.
20. For potential misclassification of intrapartum stillbirth and early neonatal death, any remedial efforts to address this issue should be mentioned.
21. In addition to the limitations provided, mention should also be made of possible bias and confounding and the potential implications of these for results of studies examining the main outcomes.
22. A few sentences on the extent to which results from the MNHR sites might be generalizable to other areas should be added, or if this is covered in other supplement papers, this should be briefly mentioned.

List of abbreviations:

23. Add abbreviation for "Traditional birth attendant" and move to its own line; add GN and NIH and ensure these are spelled out at first use in the text.

Figures and tables:

24. Figure 1: Switch to portrait for ease in viewing (as required by journal guidelines). Label the two sites in India separately.
25. Table 1: Spell out "PI".
26. Table 2: Add "(%)" to row header "Delivery, N". Clarify % of what for both this row and 42-day follow-up. Add % enrollment (in row headed "pregnant women") and remove the phrase "data not shown" from Results. Preferably break down by year. Switch to portrait for ease in viewing.

Discretionary revisions:

Background ("Introduction"):

1. (parag. 3) Add the year that the FIRST BREATH study was completed.
2. (parag. 4) The sentence beginning "For topics of specific interest…" seems out of place here; consider moving to the prior paragraph describing the purposes of the MNHR.
3. (parag. 4) The final sentence explaining the replacement of Argentina with the DRC should explain why re-competition happened and then the sentence should be moved to an appropriate place in the Methods section.
4. A few sentences of description on how data from the MNHR may have been used to influence public health policy and improve public health outcomes in the first years (2008-2009), prior to the period this paper covers, would provide useful context for the reader and emphasize the importance and uses of the MNHR.
5. The Conclusion states that the MNHR is "among the first" multicenter population-based pregnancy outcomes registries. A brief overview of other relevant registries and how the MNHR relates to them would also provide useful context for the reader.

Methods (multiple sections):

6. The bullet-point list of settings would be better presented in table format. The list and its introductory paragraph could be placed under a sub-heading of Setting.
7. The detailed description of registry staffing (parags beginning "A MNHR study committee…" and "Each cluster employs…") would be easier to understand if presented as an organization chart including a divided cell for each position listing the titles and duties of each position or entity separately.
8. Within the section currently headed "Methods", the paragraph starting "Annually, and in response to…" (and bullet point list) could be switched with the paragraph starting "Data are recorded…" for a more logical flow.
9. Making the bullet point list of variables comprehensive would help researchers consider how MNHR data might be used in their own contexts. Prolonged labor, obstructed labor, failure to progress, breastfeeding onset, contraceptive use, and other variables that are currently or have been collected should be added to this list, which might be easier for the reader to understand in a text box format. If stillbirth causes have been collected, this should also be stated.
10. Definitions for all terms in this list should be provided in the paragraph starting "The MNHR describes populations…", eg low birth weight and causes of maternal and neonatal deaths.
11. The parag on Training should be combined with the parag on data collection (which starts "Data are recorded at …"). If not described elsewhere in the supplement, this would be a good place to mention retention and turnover of trained staff.
12. A reorganization of this section and some changes in the sub-headings could help the reader navigate. One option is sub-headings titled Design, Setting, Participants, Outcomes and risk factors, Organization & management, Data collection, Sample size and power.

Results ("Enrollment in the MNHR 2010-2013"):

13. The sentence beginning "Since the inception…" should state the year of inception.

Discussion and conclusions ("Limitations" and "Conclusion"):

14. The limitations section covers three separate topics that might be better presented as three separate paragraphs.
15. The first limitation, how to ensure inclusion of all pregnancies, is split into two parts that could be brought together (sentences beginning "One of the limitations of the MNHR is the difficulty in…" and beginning "In addition, some sites have faced challenges in tracking…").
16. Implications of the decision to replace the study site in Argentina with one in the DRC should be briefly mentioned.
17. Brief mention should be made of other recent studies of pregnancy outcomes in the MNHR sites over the 2010-2013 period (including any routine government data) and how the MNHR results help fill gaps in knowledge.
18. A sentence comparing coverage data from this paper (2010-2013) with data from 2008-2009, if available, would help solidify the reader's understanding of how this paper fits within the MNHR and help contextualize the coverage results.
19. To increase the paper's utility for researchers from low-resource countries, it would be helpful to mention plans for the MNHR's future, including whether funding is secured for continuation, and if so, for how many years; and how the MNHR relates to national health plans, policies and ministries in each host country.
20. To better highlight the MNHR's impact, it would be helpful to mention any specific progress in public policy and/or pregnancy outcomes that might be related to the MNHR in the 2010-2013 period. If this is covered in other papers in the supplement, that shod be stated here.
